# Conserved and Plant-Unique Mechanisms Regulating Plant Post-Golgi Traffic

**DOI:** 10.3389/fpls.2012.00197

**Published:** 2012-08-28

**Authors:** Masaru Fujimoto, Takashi Ueda

**Affiliations:** ^1^Department of Biological Sciences, Graduate School of Science, The University of TokyoTokyo, Japan; ^2^Japan Science and Technology Agency, Precursory Research for Embryonic Science and TechnologyKawaguchi, Japan

**Keywords:** coat protein complex, dynamin-related protein, membrane trafficking, Rab GTPase, tether, SNARE

## Abstract

Membrane traffic plays crucial roles in diverse aspects of cellular and organelle functions in eukaryotic cells. Molecular machineries regulating each step of membrane traffic including the formation, tethering, and fusion of membrane carriers are largely conserved among various organisms, which suggests that the framework of membrane traffic is commonly shared among eukaryotic lineages. However, in addition to the common components, each organism has also acquired lineage-specific regulatory molecules that may be associated with the lineage-specific diversification of membrane trafficking events. In plants, comparative genomic analyses also indicate that some key machineries of membrane traffic are significantly and specifically diversified. In this review, we summarize recent progress regarding plant-unique regulatory mechanisms for membrane traffic, with a special focus on vesicle formation and fusion components in the post-Golgi trafficking pathway.

## Introduction

**Figure 1:**
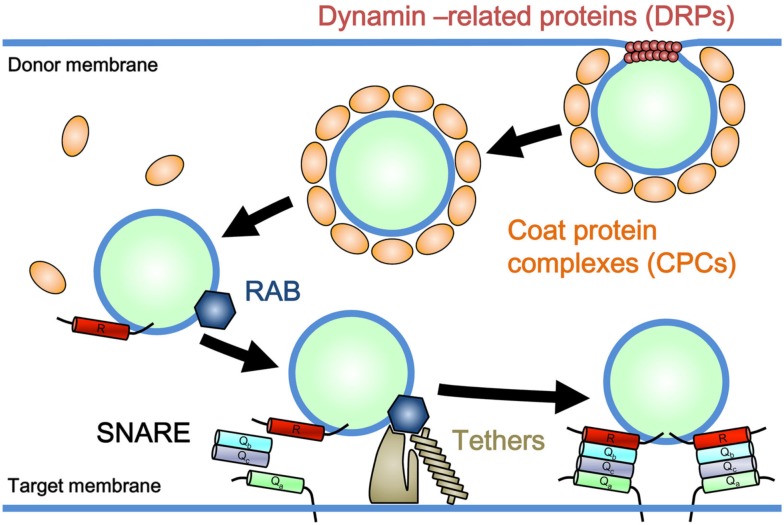
**Model for a general mechanism of membrane trafficking**. Coat protein complexes (CPCs) and dynamin-related GTPases (DRPs) participate in the formation of vesicular or tubular carriers. CPCs facilitate cargo selection and membrane deformation, and DRPs take part in the tubulation and/or scission of donor membranes. Rab GTPase promotes the tethering of membrane carriers to the target membrane through effector molecules (Tethers), which is followed by SNARE-mediated membrane fusion.

Eukaryotic cells are distinguished by the presence of internal membrane-bound organelles, including mitochondria, plastids, peroxisomes, and other single membrane-bound organelles. Two different underlying mechanisms have been proposed for the emergence of these organelles. Mitochondria and plastids, which have a double-membrane envelope in principle, arose respectively through the symbiotic incorporation of α-proteobacteria and cyanobacteria by the ancestral eukaryotic cell (Barbrook et al., 2006; Embley and Martin, 2006). In contrast, organelles bound by a single membrane layer [e.g., the endoplasmic reticulum (ER), Golgi apparatus, *trans*-Golgi network (TGN), plasma membrane (PM), and a series of endosomal compartments], are thought to have evolved autogenously from preexisting single membrane components in ancient proto-eukaryotic cells (Cavalier-Smith, 1987, 2002). These single membrane-bound organelles are connected with each other through a trafficking system mediated by vesicular and/or tubular membranous transport carriers, known as membrane trafficking. Membrane trafficking consists of several sequential processes: the formation of cargo-bearing vesicles or tubules from donor membranes, targeted delivery of transport carriers, and tethering of carriers to target membranes, followed by membrane fusion (Figure 1). These processes involve specific sets of regulatory machinery. For example, coat protein complexes (CPCs) and dynamin-related GTPases (DRPs) participate in the formation of vesicular or tubular carriers; CPCs facilitate cargo selection and membrane deformation, and DRPs take part in the tubulation and/or scission of donor membranes (Figure 1; Bonifacino and Glick, 2004; Praefcke and McMahon, 2004). Rab GTPase, a member of the Ras superfamily, tethers, and soluble *N*-ethylmaleimide-sensitive factor attachment protein receptors (SNAREs) are responsible for the targeting and subsequent tethering and fusion of carriers to target membranes (Figure 1; Chen and Scheller, 2001; Seabra and Wasmeier, 2004; Yu and Hughson, 2010). Each subfamily of these machinery components performs a function similar to that of other paralogs, but at a specific subcellular location or as part of a distinct transport pathway (Bonifacino and Glick, 2004).

Modern phylogenetic studies suggest that eukaryotes are comprised of five major supergroups: Amoebozoa; Opisthokonta; Archaeplastida; Excavata; and *St*ramenopiles, *Al*veolates and *R*hizaria together with *C*ryptophyte, *C*entrohelid, *T*elonemid, and *H*aptophyte (SAR + CCTH; Burki et al., 2009). Owing to the recent accumulation of genomic resources among these five supergroups, molecular evolutionary analyses have been yielding information about the emergence and establishment of membrane trafficking components. Comparative genomic and phylogenetic analyses of CPCs (Schledzewski et al., 1999), DRPs (Elde et al., 2005; Miyagishima et al., 2008), Rab GTPases (Brighouse et al., 2010; Elias et al., 2012), tethers (Koumandou et al., 2007), and SNAREs (Dacks and Doolittle, 2002; Yoshizawa et al., 2006) sampled from a broad range of eukaryotic lineages have revealed that similar sets of paralogous subgroups of these machinery components are often shared among these supergroups. This conservation suggests that the basic framework of membrane traffic was already established before the last common eukaryotic ancestor, which is commonly shared among extant descendant eukaryotic lineages (Dacks et al., 2009). However, these analyses also demonstrated that each eukaryotic lineage occasionally acquired lineage-specific new subgroups and/or expanded specific subfamilies, probably by lineage-specific gene multiplication and the accumulation of mutations. Such genes are predicted to be associated with the lineage-specific differentiation of organelle functions and membrane trafficking pathways.

The lineage-specific expansion and diversification of machinery components for membrane trafficking are also evident in plant lineages, especially in land plants (Embryophytes). For example, some specific Rab GTPase, component of tethering factors, and SNARE subfamilies are remarkably expanded and functionally diversified (Vernoud et al., 2003; Sanderfoot, 2007; Woollard and Moore, 2008; Saito and Ueda, 2009; Chong et al., 2010). Moreover, plant-unique molecular machineries, which are structurally distinct from closely related homologs conserved in a wide range of eukaryotic organisms, take part in fundamental membrane trafficking processes in plant cells (Ebine et al., 2008, 2011; Fujimoto et al., 2010; Van Damme et al., 2011). This review summarizes recent advances in the study of plant post-Golgi trafficking pathways, focusing on unique aspects of the plant system.

## Coat Protein Complexes

A single round of trafficking between two organelles begin with the formation of transport vesicles from the donor organelle (Figure 1). In this process, cytosolic CPCs perform pivotal roles in membrane deformation and cargo recruitment. Three classes of CPCs are widely utilized in a range of eukaryotic organisms (Schledzewski et al., 1999; Singh and Gupta, 2004; Elde et al., 2005; Dacks and Field, 2007): coat protein complex II (COPII) mediates ER-to-Golgi trafficking, coat protein complex I (COPI) mediates intra-Golgi and Golgi-to-ER trafficking (Lee et al., 2004), and clathrin-based complexes are involved in multiple steps in post-Golgi trafficking (McMahon and Mills, 2004). These three CPCs are likely to have a common ancestral origin, which may be also the origin of the nuclear pore complex (Devos et al., 2004).

Clathrin-based complexes mainly comprise clathrin coats and adaptor molecules such as cargo- or lipid-binding proteins. The clathrin coat is made up of a three-legged structure called the triskelion, each leg of which consists of a heavy chain (CHC) and a light chain (CLC) (Brodsky et al., 2001). Triskelia assemble into a lattice surrounding the membrane bud on the TGN, PM, endosomes, and lysosomes/vacuoles, and concentrate adaptors bound with cargo, leading to the loading of proteins and lipids into forming vesicles (Crowther and Pearse, 1981; Hanover et al., 1984). Although the overall architecture is well conserved among clathrin coats in eukaryotic lineages, including plants (Coleman et al., 1987), the triskelion structure of the plant clathrin coat exhibits several distinct characteristics. The plant triskelion has a higher molecular mass and longer arms than the mammalian triskelion (Mersey et al., 1985; Depta and Robinson, 1986; Coleman et al., 1987; Depta et al., 1987), suggesting that unique properties were added to plant clathrin coats during evolution.

Adaptor protein (AP) complexes AP-1 through AP-5 are central organizers that mediate cargo recognition at forming vesicles in post-Golgi trafficking pathways (Pearse and Robinson, 1984; Dell’Angelica et al., 1997, 1999; Hirst et al., 1999, 2011; Robinson and Bonifacino, 2001). Each AP complex consists of four subunits called adaptins, which are large α/γ/δ/ε/ζ and β subunits, a medium μ subunit, and a small σ subunit (Boehm and Bonifacino, 2002; Hirst et al., 2011). During vesicle formation, AP complexes link the clathrin lattice and select membrane cargos and lipids. AP complexes also bind other accessory proteins, which in turn regulate the assembly and disassembly of the coat (Bonifacino and Traub, 2003). All of these AP complexes are observed among all eukaryotic lineages with sporadic secondary loss in some clades, indicating an ancient origin for all five complexes (Hirst et al., 2011). Currently, each AP complex is assigned to a distinctive location and function: bi-directional trafficking between endosomes and the TGN for AP-1 (Boehm and Bonifacino, 2002; Robinson et al., 2010), endocytosis from the PM for AP-2 (Bar et al., 2009; Jackson et al., 2010), traffic from early endosomes/TGN to late endosomes and lysosomes/vacuoles for AP-3 (Dell’Angelica, 2009; Niihama et al., 2009; Feraru et al., 2010), TGN-to-endosome trafficking for AP-4 (Burgos et al., 2010), and trafficking around the late endosomes for AP-5 (Hirst et al., 2011). Among these AP complexes, AP-1 and AP-2 have been demonstrated to interact with clathrin, while other complexes are thought to be able to act without associating with clathrin. The ancient origin of the AP complexes suggests that their functions are also conserved in plants, which should be verified in future studies.

In addition to the conserved AP complexes, land plants have a unique adaptor-like protein, TPLATE, which contains a domain similar to β-adaptin and interacts with clathrin (Van Damme et al., 2006, 2011). TPLATE is specifically targeted to the expanding cell plate and the particular region of the PM around the site of fusion between the expanding cell plate and the mother cell, to which clathrin is also localized (Van Damme et al., 2011). Thus, TPLATE is expected to act in clathrin-mediated endocytosis during cell plate formation. Restriction of the lateral diffusion of KNOLLE, a SNARE protein, at the PM of mother cells during cytokinesis and its removal from the PM are accomplished by clathrin-mediated endocytosis (Segui-Simarro et al., 2004; Boutte et al., 2010). KNOLLE is a possible cargo of TPLATE-mediated endocytosis.

## Dynamin-Related Proteins

Dynamin-related proteins (DRPs) are large GTPases that regulate membrane fission, fusion, and tubulation during diverse cellular activities such as endocytosis, cytokinesis, vacuolar sorting, fission and fusion of mitochondria, biogenesis of peroxisomes, and the maintenance of ER morphology (Praefcke and McMahon, 2004; Hu et al., 2009). In many cases, distinct DRP proteins are assigned to fulfill different cellular functions. However, in *Trypanosoma brucei*, a protist belonging to the Excavata supergroup, a single DRP mediates both mitochondrial division and post-Golgi trafficking, including endocytosis (Chanez et al., 2006). By contrast, in the SAR + CCTH supergroup, the DRP protein that acts in endomembrane trafficking has not been found thus far, while DRPs acting in mitochondrial and/or plastid divisions have been identified in this supergroup (Miyagishima et al., 2008; van Dooren et al., 2009). These lines of evidence might suggest that an ancient function of DRPs was the regulation of membrane remodeling associated with mitochondrial endosymbiosis, although it is also plausible that DRPs for membrane trafficking were secondarily lost during evolution of the SAR + CCTH lineage. It has also been reported that some bacterial species possess DRP-like proteins that are able to deform lipid bilayers, implying a possible prokaryotic origin of this protein family (Low and Lowe, 2006; Burmann et al., 2011).

Among DRP family members, dynamin is the best-characterized member that acts in clathrin-mediated trafficking in animal cells (Sever, 2002). During clathrin-coated vesicle (CCV) formation, dynamin assembles into helical or ring-shaped structures at the neck of clathrin-coated buds (Takei et al., 1995), and constricts, severing the bud neck membrane in a GTP hydrolysis-dependent manner (Sweitzer and Hinshaw, 1998; Macia et al., 2006). Animal dynamin contains five distinct domains: the *N*-terminal GTPase domain; a middle domain that mediates intermolecular interaction during self-assembly; a GTPase-effector domain (GED), which stimulates the GTPase activity required to enact structural change in a dynamin polymer; a pleckstrin homology (PH) domain, which mediates binding to the membrane phosphoinositide; and a proline-rich domain (PRD), which is required for the recruitment of dynamin to clathrin-coated pits (Heymann and Hinshaw, 2009). The former three domains are conserved among almost all DRP proteins. DRPs with a domain configuration similar to that of dynamin, which also harbor the two additional domains, have been observed only in metazoa and land plants (Chanez et al., 2006; Miyagishima et al., 2008; Heymann and Hinshaw, 2009).

**Figure 2:**
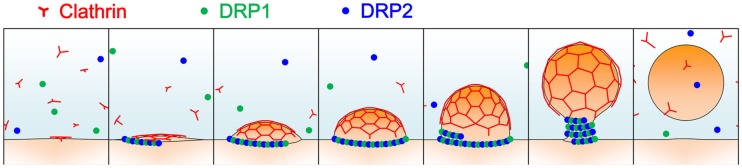
**A schematic illustration of clathrin-coated vesicle formation in land plants**. Light blue represents the cytosol; orange, and red lines represent the donor membrane and the clathrin coat, respectively; and green and blue dots represent DRP1 and DRP2 proteins, respectively.

Most land plants’ genomes contain six types of DRPs: DRP1-DRP4, DRP5A, and DRP5B (Hong et al., 2003; Miyagishima et al., 2008). Of these subfamilies, two structurally different DRPs, DRP1, and DRP2, are involved in clathrin-dependent trafficking events including endocytosis and cell plate formation (Figure 2; Hong et al., 2003; Kang et al., 2003; Collings et al., 2008; Fujimoto et al., 2008, 2010; Konopka et al., 2008; Taylor, 2011). DRP2 shares overall domain organization with animal dynamin, while DRP1 lacks the PH domain and PRD; a DRP with a similar structure is only found in green plants (Hong et al., 2003). In spite of the similarity in the overall domain structure between plant DRP2 and animal dynamin, the GTPase domain of animal dynamin exhibits greater similarity to the GTPase domains of DRP1 members than to that of DRP2 (e.g., 66% identity to *A. thaliana* DRP1A, and 27% identity to *A. thaliana* DRP2B). These lines of evidence raise the possibility that complementary functions of plant DRP2 and DRP1 are required to fulfill the function in clathrin-mediated trafficking events, which animal dynamin executes by itself. In a consistent manner, these two subfamilies of DRPs interact with each other and assemble together with clathrin at discrete foci at the PM (Fujimoto et al., 2010). Cooperative action of two structurally distinct DRPs in the same membrane scission event has not been reported in other organisms; thus, plants appear to have developed a unique mechanism for endocytic vesicle formation.

## Rab GTPases

Rab GTPases, which comprise the largest family in the Ras superfamily, act as molecular switches to regulate the targeting and tethering of transport carriers to target membranes by cycling between GTP-bound active and GDP-bound inactive states (Figure 1; Saito and Ueda, 2009). The activation of Rab GTPases occurs with the exchange of bound GDP for GTP, which is catalyzed by guanine nucleotide exchange factor (GEF). GTP-bound Rab GTPases interact with specific effector molecules that evoke downstream reactions including the tethering of transport carriers to target membranes by tethers (Grosshans et al., 2006). Tethering between two membranes is mediated by a group of multi-subunit complexes (e.g., HOPS, TRAPP, and Exocyst) and/or long fibrous proteins (e.g., EEA1 and p115/Uso1p), most of which act as effectors of Rab GTPases (Cai et al., 2007; Markgraf et al., 2007). Because Rab GTPases exhibit a much greater degree of phylogenetic diversification than other tethering components, they are thought to be vital players in the diversification of the endomembrane system (Dacks and Field, 2007; Gurkan et al., 2007; Elias, 2010). Recent comprehensive genomic analysis has suggested that the last common eukaryotic ancestor harbored at least 23 groups of Rab GTPases (Elias et al., 2012) – substantially more than have been found in many extant eukaryotic organisms, including plants. Thus, the secondary loss of Rab GTPases (and the acquisition of new ones) occurred in a wide range of eukaryotes during evolution.

Plant Rab GTPases also appear to have followed a unique path of diversification and evolution. The genome of *A. thaliana* contains 57 Rab GTPases that are classified into eight groups (RABA–RABH). Each group exhibits a high degree of similarity to animal RAB1, RAB2, RAB5, RAB6, RAB7, RAB8, RAB11, or RAB18 (Rutherford and Moore, 2002; Vernoud et al., 2003). Most land plants possess these eight groups in theory, with a few additional members of unknown function in basal land plants (Rensing et al., 2008; Banks et al., 2011). Compared with other eukaryotic lineages, one distinct feature of the land plant Rab GTPase is extreme expansion of the RABA/RAB11 group (Rutherford and Moore, 2002). In *A. thaliana*, 26 of 57 Rab GTPases belong to this group, which are further divided into six subgroups, RABA1 through RABA6 (Rutherford and Moore, 2002). In contrast, three of 66 and two of 11 RAB GTPases in *Homo sapiens* and *Saccharomyces cerevisiae*, respectively, are members of the RAB11 group (Pereira-Leal and Seabra, 2001; Stenmark and Olkkonen, 2001).

Yeast and animal RAB11 members function at multiple steps of post-Golgi trafficking pathways (Benli et al., 1996; Ullrich et al., 1996; Jedd et al., 1997; Chen et al., 1998; Strickland and Burgess, 2004). In addition, in land plants, RABA members have been shown to localize around the TGN (Ueda et al., 1996; de Graaf et al., 2005; Chow et al., 2008; Szumlanski and Nielsen, 2009), which also acts as the early endosome in plant cells (Dettmer et al., 2006; Viotti et al., 2010). The diversity of RABA/RAB11 members in land plants suggests that plant-unique functions are assigned to members of this group, some of which are demonstrated by recent studies. For example, RABA2 and A3 are involved in cell plate formation (Chow et al., 2008), and RABA1 and RABA4 are required for normal tip growth of pollen tubes and root hairs (Preuss et al., 2004; de Graaf et al., 2005; Szumlanski and Nielsen, 2009). It has also been proposed that the function of RABA is associated with the biogenesis and degradation of the cell wall during fruit development (Zainal et al., 1996; Lu et al., 2001; Abbal et al., 2008; Lycett, 2008).

The diversification of the RAB5 group is another distinctive feature of the plant Rab GTPase. RAB5 is a broadly conserved Rab GTPase, and regulates a wide range of early endocytic trafficking in animal cells (Somsel Rodman and Wandinger-Ness, 2000; Benmerah, 2004). Orthologs of animal *RAB5* are also conserved in all plant species whose genomes have been sequenced thus far, with the exception of a unicellular rhodophyte, *Cyanidioschyzon merolae* (Matsuzaki et al., 2004). Land plants also harbor another plant-unique type of RAB5 molecule, the ARA6/RABF1 group, which is structurally distinct from conventional RAB5 (Ebine and Ueda, 2009). The *A. thaliana* genome has three RAB5-related genes: two conventional type *RAB5*, *RHA1*/*RABF2a*, and *ARA7*/*RABF2b*; and one plant-unique *RAB5*, *ARA6*/*RABF1* (Ueda et al., 2001). All of the three RAB5s in *A. thaliana* have been detected on multivesicular endosomes (MVEs) using electron microscopy (Haas et al., 2007), and are activated by the same GEF, VPS9a (Goh et al., 2007). However, the subcellular localizations of two types of RAB5 did not overlap completely when their localization was compared in the same cell (Ueda et al., 2004), and the overexpression of constitutively active ARA6 and ARA7 conferred different effects on a partial loss-of-function mutant of *VPS9a*, *vps9a-2* (Goh et al., 2007).

Recently, we have successfully demonstrated the functional diversification between these conventional and plant-unique types of RAB5 in *A. thaliana*. Genetic, biochemical, and imaging analyses indicated that ARA6 acts in the trafficking pathway from MVEs to the PM, while conventional RAB5 is involved in the trafficking pathway between MVEs and vacuoles (Ebine et al., 2011). The *ara6* mutation resulted in hypersensitivity to salinity and osmotic stresses, and overexpression of constitutive active ARA6 conferred salinity stress tolerance to *A. thaliana* plants (Ebine et al., 2011, 2012). Possible involvement of *ARA6* homologs in the stress response has also been reported for other land plant species (Bolte et al., 2000; Zhang et al., 2009). Considering with that *ARA6* homologs are well conserved among land plants and rather sporadic in algal lineages, it may be adaptive for land plants to retain the trafficking pathway involving the ARA6-type RAB5 for survival in terrestrial conditions.

Another class of small GTPase, Rho-like GTPases of plants (ROP), has recently been demonstrated to have regulatory roles in plant membrane trafficking, which is required for the auxin-mediated establishment of cell polarity (Chen et al., 2012; Lin et al., 2012; Nagawa et al., 2012). Directional transport of auxin depends on a family of auxin efflux carriers known as PIN-FORMED (PIN) proteins, which undergo constitutive endocytic recycling (Dhonukshe et al., 2007, 2008; Kleine-Vehn et al., 2011). In the roots, auxin affects its own transport by inhibiting the clathrin-mediated endocytosis of PIN1 and PIN2, which is mediated by Auxin Binding Protein 1 (ABP1; Paciorek et al., 2005; Robert et al., 2010), as well as the downstream signaling molecules ROP6 and ROP-interactive CRIB motif containing protein 1 (RIC1; Chen et al., 2012; Lin et al., 2012). In addition, ROP and RIC have been shown to act in the morphogenesis of leaf epidermal cells. Auxin and ABP1 promote interdigitation of epidermal pavement cells by activating a signaling pathway involving ROP2 and RIC4 (Fu et al., 2005; Xu et al., 2010), which causes the accumulation of cortical actin microfilaments, thereby resulting in the local inhibition of clathrin-dependent endocytosis and asymmetric distribution of PIN1 (Nagawa et al., 2012). Rho GTPase-mediated inhibition of endocytosis is also centrally involved in the establishment of cell polarity in animal systems (Izumi et al., 2004; Harris and Tepass, 2008). Thus, the plant appears to have developed its multicellular body plan by recruiting the common mechanism of polarity regulation, as well as by adding plant-specific innovations such as ABP1 and RICs (Napier et al., 2002; Nagawa et al., 2010).

## Tethers

Some of proteins that mediate the tethering of transport vesicles to target membranes also appear to be diversified in a unique way in plants. A comparative genomic analysis suggested that each tethering complex has an ancient and independent origin; a set of non-homologous tethering complexes is conserved across all eukaryotic lineages, with frequent secondary losses (Koumandou et al., 2007). Acquisition of the non-homologous tethering complexes may be the other driving force for the diversification of membrane trafficking pathways, in addition to the paralogous expansion observed in the Rab and SNARE families.

While the obvious orthologs of long fibrous tethers have not been found, the components of tethering complexes are well conserved in plants (Koumandou et al., 2007). In *A. thaliana*, components of the HOPS/CORVET complexes residing on vacuolar membranes and pre-vacuolar compartments are involved in vacuolar biogenesis and transport to vacuoles (Rojo et al., 2001, 2003; Niihama et al., 2009). The TRAPP complex, which is further divided into TRAPPI and TRAPPII, is required for cell plate formation (Thellmann et al., 2010). The importance of the exocyst complex in various secretion-related events, including tip growth of pollen tubes and root hairs, hypocotyl elongation, deposition of seed coat pectin, pollen acceptance at the stigma, and pathogen responses, has also been reported (Cole et al., 2005; Synek et al., 2006; Hala et al., 2008; Chong et al., 2010; Kulich et al., 2010; Pecenkova et al., 2011). The GARP complex is also involved in pollen tube growth (Lobstein et al., 2004; Guermonprez et al., 2008), as well as resistance to heat and osmotic stresses (Lee et al., 2006).

Among the tethering complexes in land plants, the exocyst complex appears to be assigned to uniquely diversified functions in exocytosis-related events. The exocyst consists of eight evolutionarily conserved subunits, SEC3, SEC5, SEC6, SEC8, SEC10, SEC15, EXO70, and EXO84, whose assembly mediates the tethering of secretory vesicles to the target PM during the last step of exocytosis (Munson and Novick, 2006). Among the exocyst subunits, the EXO70 family exhibits remarkable expansion in land plants. In contrast to a single copy of the *EXO70* gene in most of opisthokonta genomes, multiple *EXO70* genes have been observed in a variety of land plant genomes: 13 in *Physcomitrella patens*; 23 in *A. thaliana* and *Populus trichocarpa*; and 41 in *Oryza sativa* (Chong et al., 2010), which can be divided into three families comprising nine subfamilies (Elias et al., 2003; Synek et al., 2006; Chong et al., 2010).

Although the functions of the majority of EXO70 family proteins remain unknown, several *A. thaliana* EXO70 members are localized to endosomal compartments including the TGN/early endosome (Chong et al., 2010). Recently, a unique function of an *A. thaliana* EXO70 family member, Exo70E2, has been reported. EXO70E2 was localized to spherical double-membrane structures resembling autophagosomes and were named exocyst-positive organelles (EXPOs; Wang et al., 2010). Intriguingly, standard markers for conventional organelles (including the Golgi apparatus; TGN and early endosomes; MVE/late endosomes; and autophagosomes) did not occur on EXPOs (Wang et al., 2010), and brefeldin A and wortmannin did not affect EXPO distribution. EXPOs have been suggested to mediate a form of unconventional protein secretion unique to land plants: the transport of cytosolic proteins to the cell exterior (Wang et al., 2010). Additional studies of other EXO70 family members, as well as functional analyses of other exocyst components, would lead to understanding the unique and diverse exocytic mechanisms that plants have acquired during the course of evolution.

## SNARE Family Proteins

At the final step of a single round of trafficking, SNARE family proteins, which are evolutionarily conserved integral or peripheral membrane proteins, execute membrane fusion between transport carriers and target membranes (Figure 1; Jahn and Scheller, 2006; Wickner and Schekman, 2008; Saito and Ueda, 2009). The SNARE family consists of four subgroups, Qa-, Qb-, Qc-, and R-SNAREs, which are classified according to the presence of a conserved glutamine (Q) or arginine (R) residue in a particular helical domain called the SNARE domain. In general, Q- and R-SNAREs reside on distinct membrane compartments, and three Q-SNAREs (Qa, Qb, and Qc) and an R-SNARE assemble into a tight complex in specific combinations, leading to membrane fusion between two compartments. Most SNARE proteins, except for SNAP-25-like members, contain one SNARE domain in their polypeptides (Jahn and Scheller, 2006). Recent comprehensive genomic analyses have indicated that a significant increase in the number of SNARE family members occurred with the acquisition of developmental complexity – for example, from unicellular to multicellular organisms (there are 17 SNAREs in *C. merolae*, 24 in *S. cerevisiae*, 26 in *Chlamydomonas reinhardtii*, 38 in *H. sapiens*, and 63 in *A. thaliana*; Dacks and Doolittle, 2002; Yoshizawa et al., 2006; Sanderfoot, 2007; Dacks et al., 2008). This increase is in good agreement with the hypothesis that multiplication followed by the functional diversification of key components of membrane trafficking, including SNAREs, is a prerequisite for the diversification of membrane trafficking pathways (Dacks and Doolittle, 2002; Yoshizawa et al., 2006; Dacks and Field, 2007; Dacks et al., 2008), which in turn should be required to support increasingly complex body plans and life cycles.

One remarkable feature of the post-Golgi SNARE in land plants is the functional diversification of the PM-localized Qa-SNARE, the SYP1 group, which consists of nine members in *A. thaliana*. Phylogenetic relationships to animal and fungal orthologs suggest that SYP1 group members are involved in membrane fusion at the PM (Sanderfoot, 2007); some members of SYP1 group have been reported to perform specialized functions at the PM. For example, SYP111/KNOLLE is expressed in mitotic cells and plays an essential role in membrane fusion at forming cell plates during cytokinesis (Lukowitz et al., 1996; Lauber et al., 1997). SYP121/PEN1/SYR1, another SYP1 member, takes part in K^+^ uptake through the control of K^+^ channel gating (Honsbein et al., 2009; Grefen et al., 2010). This protein also participates in the non-host defense response against attack by fungal pathogens (Collins et al., 2003; Assaad et al., 2004). SYP132 appears to be centrally involved in bacterial infection and symbiosis: the silencing of a SYP132 ortholog in *Nicotiana benthamiana* resulted in impaired multiple responses against bacterial pathogens (Kalde et al., 2007), and an ortholog of SYP132 in *Medicago truncatula* localizes to the PM surrounding infection threads and the infection droplet membrane (Catalano et al., 2007).

In addition to the functional diversification of SYP1 group members, another distinct feature of post-Golgi SNAREs in land plants is the expansion of the VAMP7 R-SNARE group (Sanderfoot, 2007; Ebine and Ueda, 2009). R-SNAREs are divided into two groups, longins and brevins; longins contain an N-terminal longin domain, while brevins lack this domain (Filippini et al., 2001). Land plants harbor only longin-type R-SNARE members, which are further classified into three major groups: VAMP7, YKT6, and SEC22. While vertebrates have only one or a few VAMP7 proteins that participate in secretory and endocytic trafficking (Chaineau et al., 2009), the VAMP7 group of *A. thaliana* consists of 12 members, which are further divided into three subgroups: VAMP71, VAMP72, and VAMP727 (Uemura et al., 2004).

A phylogenic analysis has suggested that VAMP71 is a prototype from which VAMP72 and VAMP727 have been derived (Sanderfoot, 2007). VAMP71 members localize to the vacuolar membrane (Uemura et al., 2004) and are involved in salt and drought stress responses (Leshem et al., 2006, 2010). VAMP72 members localize to the TGN and function in the secretory pathway, including cell plate formation (Zhang et al., 2011). Although VAMP727 exhibits high sequence similarity to other VAMP72 members, it harbors a unique structural characteristic: VAMP727 has an insertion comprising acidic amino acid clusters in the longin domain (Ebine and Ueda, 2009; Vedovato et al., 2009). This type of VAMP7 member is well conserved in seed plants; however, it has not found in lycophytes or moss thus far, indicating relatively recent emergence of this subfamily.

VAMP727 localizes on the RAB5-positive MVE/pre-vacuolar compartment, and mediates membrane fusion between the pre-vacuolar compartment and vacuolar membranes by forming a complex with Qa-SYP22/VAM3, Qb-VTI11, and Qc-SYP51 (Ebine et al., 2008). This complex is essential for the efficient transport of storage proteins to protein storage vacuoles during the process of seed maturation. Moreover, VAMP727 also mediates membrane fusion at the PM by forming a complex with Qa-SYP121/PEN1 (Ebine et al., 2011), which is under the control of the plant-unique RAB5 ARA6. These lines of evidence may indicate that the plant explored novel trafficking pathways from the MVE to the vacuole and PM by acquiring these two plant-unique molecules, leading to the current complex and unique post-Golgi trafficking network in angiosperms.

## Perspectives

Plants have elaborated a distinctive post-Golgi trafficking system through the acquisition of plant-specific machinery components of membrane trafficking, such as TPLATE, DRP1/2, ARA6, and VAMP727, as well as the functional expansion of evolutionarily conserved components, as observed for RAB11, EXO70, and SYP1 groups. However, many unsolved questions must be answered to elucidate the precise function and regulatory mechanism of the plant-unique trafficking system. For example, with what APs does TPLATE form a complex to mediate CCV formation? Does the TPLATE complex-mediated CCV formation involve DRP proteins? Why do land plants require two structurally distinct DRPs for CCV formation? Do they in fact polymerize into the same ring- or helix-shaped structure?

Regarding the molecular machinery involved in membrane tethering and fusion, key questions also remain to be elucidated. What effector molecules mediate ARA6 function to fulfill higher-ordered functions, such as salinity stress tolerance? Is the ARA6 function revealed in *A. thaliana* shared by all ARA6 group members throughout the plant lineages? Why and how did plants expand the RAB11 group, and how are different functions of RAB11 members exerted in spite of their high sequence similarity? What is the mechanism of molecular evolution of the VAMP727 group, and what is the molecular function of the acidic insertion? Additional studies using model systems like *A. thaliana* are obviously needed to answer these questions, and would also be required to challenge distinct lineages of plants, including basal lineages (e.g., ferns, mosses, liverworts, and algae), to yield information regarding aspects of diversity and evolution of membrane trafficking. Future experimental studies employing a wide variety of plant species, together with comprehensive genomics analysis *in silico*, will help us to understand how current plant membrane trafficking pathways have evolved.

## Conflict of Interest Statement

The authors declare that the research was conducted in the absence of any commercial or financial relationships that could be construed as a potential conflict of interest.
